# A Minimal Connected Network of Transcription Factors Regulated in Human Tumors and Its Application to the Quest for Universal Cancer Biomarkers

**DOI:** 10.1371/journal.pone.0039666

**Published:** 2012-06-25

**Authors:** Ahmed Essaghir, Jean-Baptiste Demoulin

**Affiliations:** de Duve institute, Université Catholique de Louvain, Brussels, Belgium; University of Turin, Italy

## Abstract

A universal cancer biomarker candidate for diagnosis is supposed to distinguish, within a broad range of tumors, between healthy and diseased patients. Recently published studies have explored the universal usefulness of some biomarkers in human tumors. In this study, we present an integrative approach to search for potential common cancer biomarkers. Using the TFactS web-tool with a catalogue of experimentally established gene regulations, we could predict transcription factors (TFs) regulated in 305 different human cancer cell lines covering a large panel of tumor types. We also identified chromosomal regions having significant copy number variation (CNV) in these cell lines. Within the scope of TFactS catalogue, 88 TFs whose activity status were explained by their gene expressions and CNVs were identified. Their minimal connected network (MCN) of protein-protein interactions forms a significant module within the human curated TF proteome. Functional analysis of the proteins included in this MCN revealed enrichment in cancer pathways as well as inflammation. The ten most central proteins in MCN are TFs that trans-regulate 157 known genes encoding secreted and transmembrane proteins. In publicly available collections of gene expression data from 8,525 patient tissues, 86 genes were differentially regulated in cancer compared to inflammatory diseases and controls. From TCGA cancer gene expression data sets, 50 genes were significantly associated to patient survival in at least one tumor type. Enrichment analysis shows that these genes mechanistically interact in common cancer pathways. Among these cancer biomarker candidates, TFRC, MET and VEGFA are commonly amplified genes in tumors and their encoded proteins stained positive in more than 80% of malignancies from public databases. They are linked to angiogenesis and hypoxia, which are common in cancer. They could be interesting for further investigations in cancer diagnostic strategies.

## Introduction

Cancer is a multifactorial disease. Many cancer types and stages have been distinguished. This complexity makes the quest for “universal cancer biomarkers” a challenging task. However, many studies conducted separately on different cancer types have reported common genes with potential biomarker value in treatment or diagnosis [1].

On the basis of literature reviewing or by using high-throughput techniques some authors identified potential biomarkers common to several cancers and tried to develop strategies to identify them from patient biofluids either directly or indirectly. Among these markers, telomerase has been reported as being highly expressed in neoplasms [2]. A platform to capture circulating tumor cells from patient blood and measure their telomerase activity has been proposed as a cancer diagnostic tool [3]. In addition, extra-cellular cAMP-dependent protein kinase A (EC-PKA) has been reported to be a good marker for multiple cancers [4]. Auto-antibodies against EC-PKA measured by ELISA from patients sera have been found to be highly specific to cancer [5]. Follicle-stimulating hormone (FSH) receptor was also reported to be selectively expressed in a variety of tumors [6]. The same observations apply also to a cytochrome P450 (CYP1B1) [7]. Epigenetic alterations, in addition, could have a diagnostic value in cancer. Indeed, some authors have pointed to cancer-specific DNA methylation patterns as a marker for malignant diseases [8]. They can be detected on cell-free circulating DNA in the blood [9]. Auto-antibodies against leukocyte antigen F (HLA-F) were also detected in patients with various cancer types compared to healthy individuals [10].

Cancer biomarker candidate genes could be identified from literature. Confidence weights can be associated to each gene using its citation frequency [11]. Although initially used to enumerate markers specific to each cancer type, these weighted lists can help selecting common biomarkers in cancer. However, more elaborated strategies have been used to identify common cancer biomarkers, including gene expression meta-analysis across different tumor types [12], [13]. They can be associated with function and pathway annotation enrichment filters to select common biomarkers [14].

In this study, we have elaborated an integrative strategy to search for useful biomarkers common to cancer types. Our working hypothesis is based on the assumption that almost all the perturbations that lead to malignancy transformation of normal cells, although complex and diverse, share common collaborative pathways [15]. In general, these pathways might end by activating and/or repressing some sets of genes. These genes are targets of transcription factors (TFs). Some of these TFs are redundantly modulated between different cell transforming events [16]–[22]. They could be seen as connections or cross-talk nodes of the cancer leading pathways [23]–[27]. Thus, there should be a set of minimal connected TFs commonly perturbed in tumors as they share modulated pathways [28]. This set of TFs could be considered as a bottleneck of cancer pathways. If common cancer biomarkers exist, they are more likely to be among the targets of these commonly regulated TFs [29]. In this study, we took advantage of TFactS, a tool that we recently developed to predict TF regulations from high throughput gene expression data [30].

## Results

### Identification of TFs Regulated in Cancer Cell Lines

Gene expression and SNP data were available for 305 cell lines, from which results were further analyzed. These cell lines represent a broad panel of cancer types covering 28 different histological sites.

**Figure 1:**
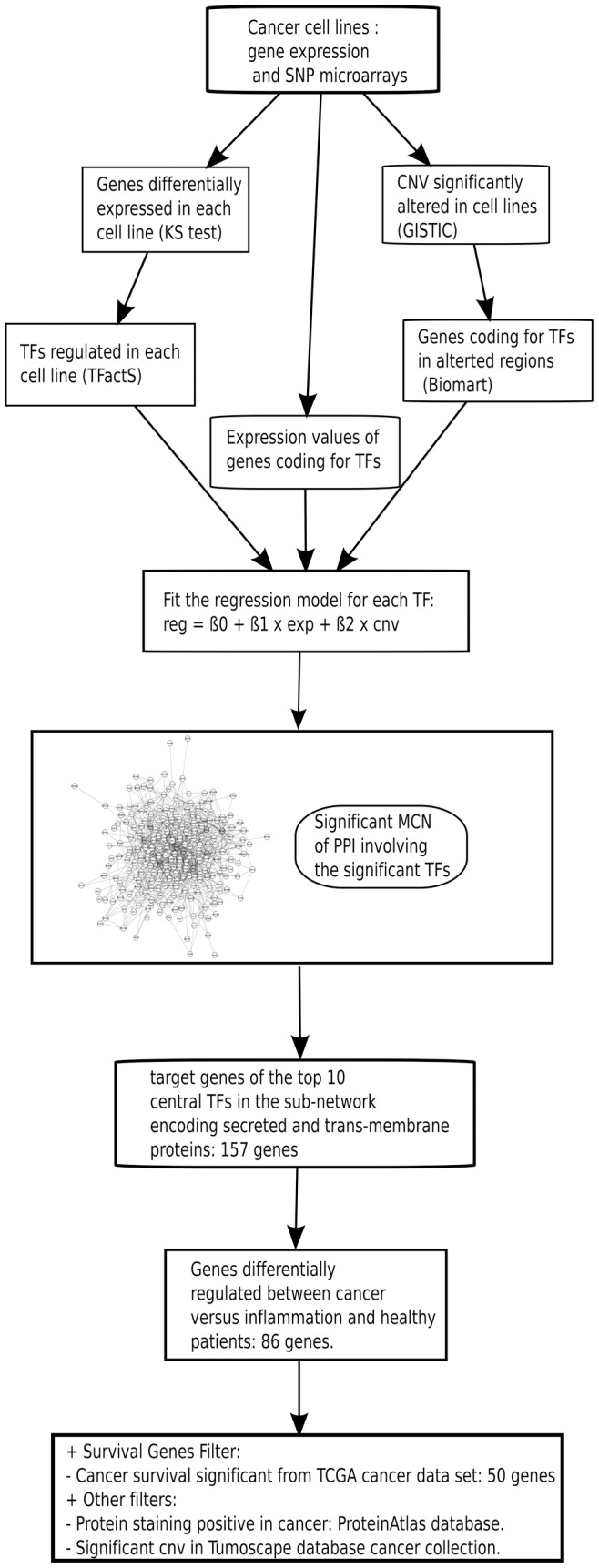
A workflow summarizing the strategy to identify accessible common cancer biomarkers. See text for details. Reg: regulation; Exp: expression; CNV: copy number variation; MCN: minimal connected network; PPI: protein-protein interactions; TF: Transcription factor.

We assumed that important TFs would be those for which gene expression and CNV could explain their activity status [31], [32]. They could be identified using the regression model shown in Figure 1. To compute all the parameters needed for this model, we identified genes differentially regulated in each cell line compared to the pool of all other cell lines. The median number of regulated genes per cell line is 218 (min: 15 and max: 721), cumulatively involving 4,686 unique known coding genes. Then, each cell line-specific gene list was submitted to TFactS and compared against catalogue of experimentally validated TF target genes using Fisher‘s test [30]. We have shown that this tool efficiently predicts TF regulation from regulated gene lists [33], [34]. On the other hand, the SNP data were normalized and segmented then submitted to the GISTIC algorithm to identify chromosomal regions significantly altered in all these cell lines [35]. Figure S1 shows that significant amplifications and deletions were spread in the whole genome. A restricted analysis of TF-encoding genes revealed that 2,113 of the 2,335 genes known to encode “DNA binding” proteins (GO term) had their loci significantly altered, at least, in one cell line. To select transcription factors relevant to cancer in a more stringent manner, we combined the analysis on expression, activity and CNV (Figure 1).

For each TF, correlation profiles with other TFs were computed based on: regulation (inferred from TFactS analysis), gene expression and genomic alterations (CNV), respectively. The model in Figure 1 uses these correlation scores to find significant TFs, for which gene expression associated to CNV could explain the corresponding inferred regulation. 88 TFs were identified (p-values < = 0.05, Table S1). Supporting our results, CNV affecting some of these TFs in cancer have already been reported, including: TP53, BRCA1, RUNX1 and MYC [36].

### The Minimal Connected Network of Transcription Factors Regulated in Cancer Cell Lines

**Figure 2:**
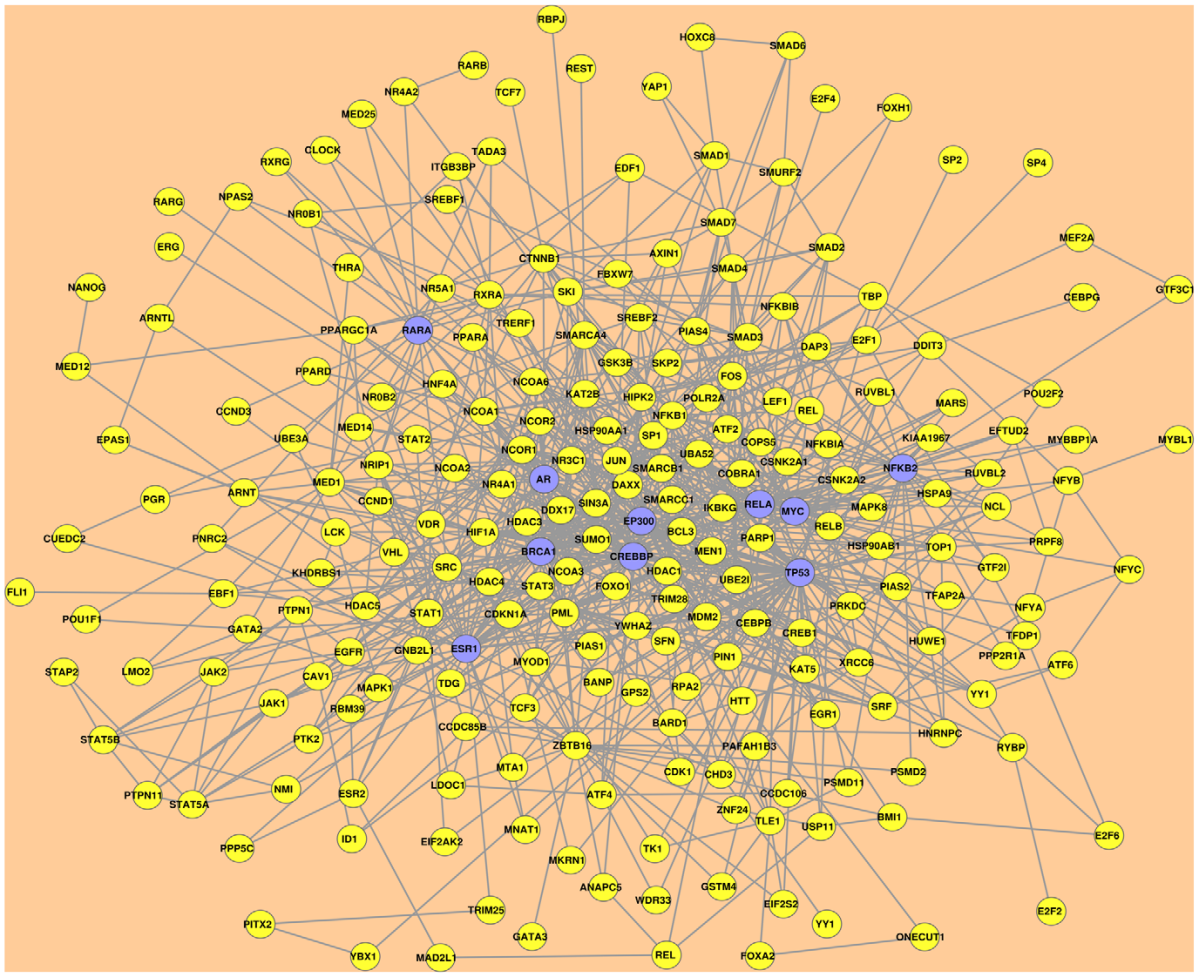
The minimal connected network of TFs regulated in cancer cell lines. The Snow web tool identified a significant human curated protein-protein interaction subnetwork involving 70 out of the 88 TFs correlatively regulated in cancer cell lines. The first connected component as shown here is considered as the minimal connected network (MCN) connecting these TFs. Each node represent a protein. Edges are the protein-protein interactions validated by at least two experimental evidences. Nodes shaded in violet represent the top ten most central TFs in the MCN. Node-ranking was based on the betweenness centrality scores.

We used the Snow web tool to identify the minimal connected network (MCN) of protein-protein interactions involving the 88 TFs associated to cancer from our initial analysis. Snow predicted this MCN by computing the shortest paths linking the input proteins either directly or with one tolerated intermediate protein, based on a built-in database of human protein-protein interactions [37], [38]. Restricting our Snow-based analysis to the human protein interactome with at least two experimental evidences of interaction, we identified a subnetwork connecting 70 out of 88 TFs either directly or with one intermediate. It is remarkable that most of the TFs identified in the first step could be linked in this single protein-protein interaction subnetwork. Eighteen TFs were lost due to our restrictions in the analysis or to their absence in the Snow-annotated interactome. Snow uses Kolmogorov-Smirnov’s test to evaluate the significance of the identified subnetwork by comparing its betweenness, connections and clustering coefficient distributions to those generated from 1,000 random networks with the same number of proteins. Our identified subnetwork had significant p-values for all these evaluated parameters (betweenness: 2.06E−37, connections: 1.68E−47, clustering coef.: 4.07E−43). This subnetwork contained two distinct connected components. The first contained almost all interactions of the significant subnetwork and was considered as the cancer cell line-associated TFs MCN for subsequent analysis (Figure 2). The second connected component, which has only two interactions linking three proteins was discarded.

We then asked whether false positives from TFactS, GISTIC and differential expression analyses could affect the MCN identification. To control these effects, we performed a negative control, in which we analyzed 100 different random lists of 88 TFs from the TFactS catalogue. Each list was submitted to Snow to produce a MCN using the same parameters as above. By comparing the distribution of the betweenness scores from all the random MCNs to the established MCN from our model, we found a significant difference (p-value ∼0.01; KS test). Together with the results discussed above from the built-in comparison with 1,000 random networks performed in Snow, this suggested that our identified MCN constitutes a significant module involving TFs commonly regulated in cancer cell lines.

**Figure 3:**
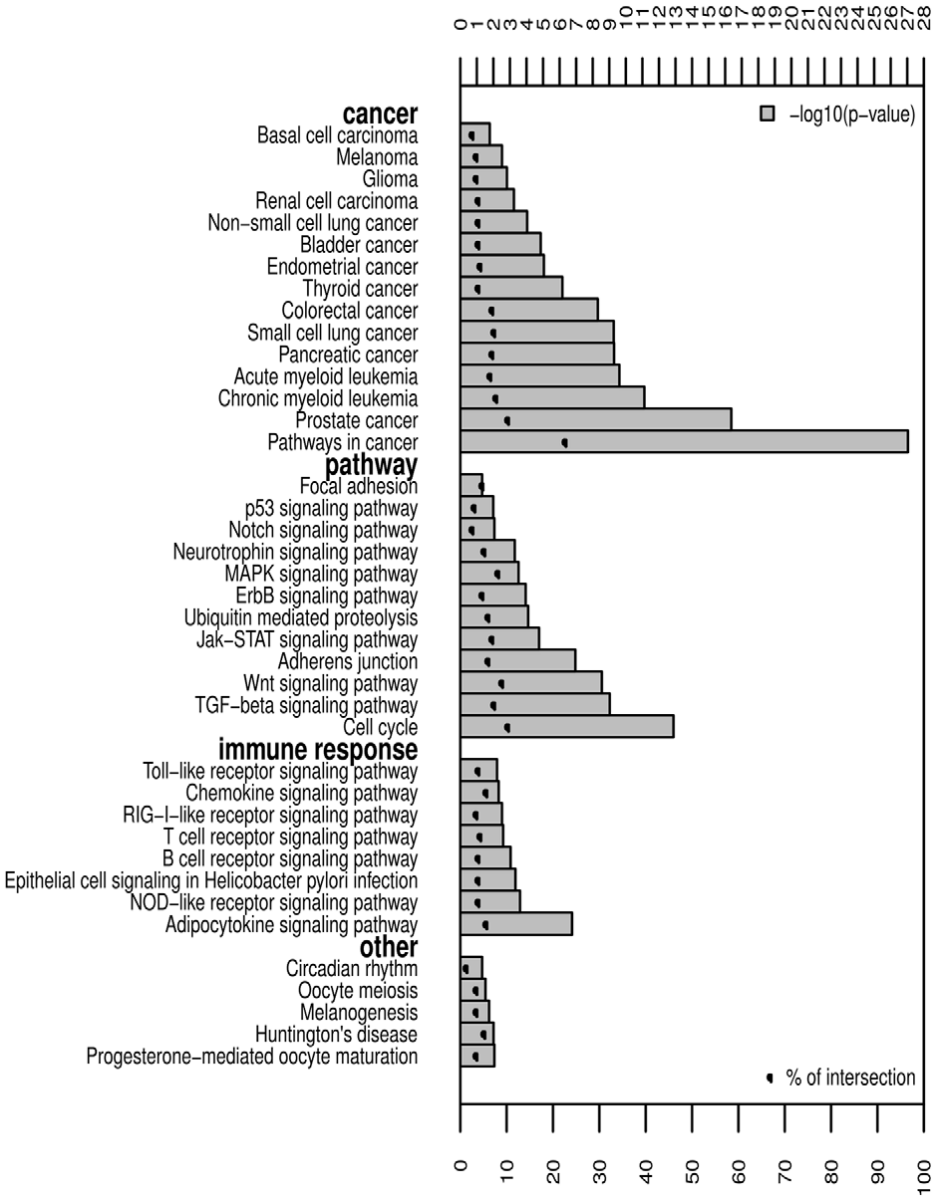
Signaling pathway enrichment in the MCN proteins. All proteins (nodes) in the MCN were submitted to DAVID web tool for KEGG pathway enrichment analysis. Significant pathways are shown by categories according to the −log10(p-value) and the percentage of intersection between the submitted list and queried annotations.

This MCN might be viewed as a regulatory “round-about” of the majority of regulated pathways in cancer cell lines. Indeed, as depicted in Figure 3, many MCN proteins are involved in many cancer types and cancer signaling pathways. Nevertheless, MCN proteins are also significantly involved in immune response pathways. This could reflect an involvement of some MCN TFs like NFkB in both cancer and inflammation [16].

### Target Genes of MCN Central Transcription Factors

Transcription factors in the minimal connected network identified above likely represent the main regulatory effectors commonly perturbed in the analyzed cancer cell lines. We focused on the most central TFs in this network. Centrality of nodes in a given network could be estimated using many parameters. Among them, betweenness scores the frequency by which a certain node is within the shortest paths linking any other two nodes. It is thought to be a good estimate of centrality [39]. By top-ranking the 236 MCN nodes according to their betweenness scores, we identified 59 central proteins having scores above the average. These central proteins show the same functional enrichment as the whole MCN.

We arbitrarily selected the top 10 central MCN nodes. Their encoding gene names are: TP53, ESR1, CREBBP, MYC, AR, BRCA1, RELA, RARA, EP300 and NFKB2. These ten TFs concentrate 41% of the total betweenness cumulative scores of the 236 MCN nodes. They could be considered as hubs or collectors of these network interactions. This is in line with the “scale free” model that was suggested to govern the TF protein-protein interactions, in which the hubs were built around TFs associated with malignancies [40]. We argued that common cancer biomarkers are likely to be found among the targets of these most central TFs. 874 unique target genes of these ten TFs are reported in the TFactS catalogue. An enrichment analysis of these genes, using “genetic association db disease” in the DAVID web tool, revealed an over-representation of a large panel of cancer types as well as ontologies related to immune responses and inflammatory diseases (File S1).

### Cancer-specific Genes from Targets of MCN Central Transcription Factors

Enrichment analysis performed on MCN proteins as well as the targets of the central TFs showed an association between cancer and inflammation. This association is well documented in literature [41]. Cancer-specific biomarkers have to be differentially expressed in cancer patients compared to healthy individuals and patients with inflammatory diseases [42]. In addition, a universal cancer biomarker should be cancer-specific in a broad panel of tumor types. Since our interest is to identify “accessible” cancer biomarkers, we sought to restrict further analysis only on genes coding for secreted and transmembrane proteins. The SP-PIR annotation keywords database, as used in the DAVID tool, contains 1,689 and 642 genes annotated as encoding secreted and transmembrane proteins, respectively. In the 874 target genes of the ten most central TFs in the MCN, we found 57 genes encoding secreted proteins (p-value: 1.1E−6) and 110 encoding transmembrane proteins (p-value: 4.3E−5). This represents a unique set of 157 genes. Thus, identifying the TFs MCN and focusing on target genes of the ten most central TFs allowed us to prioritize a short list of accessible proteins to be analyzed in patient samples for differential expression (Figure 1).

**Figure 4:**
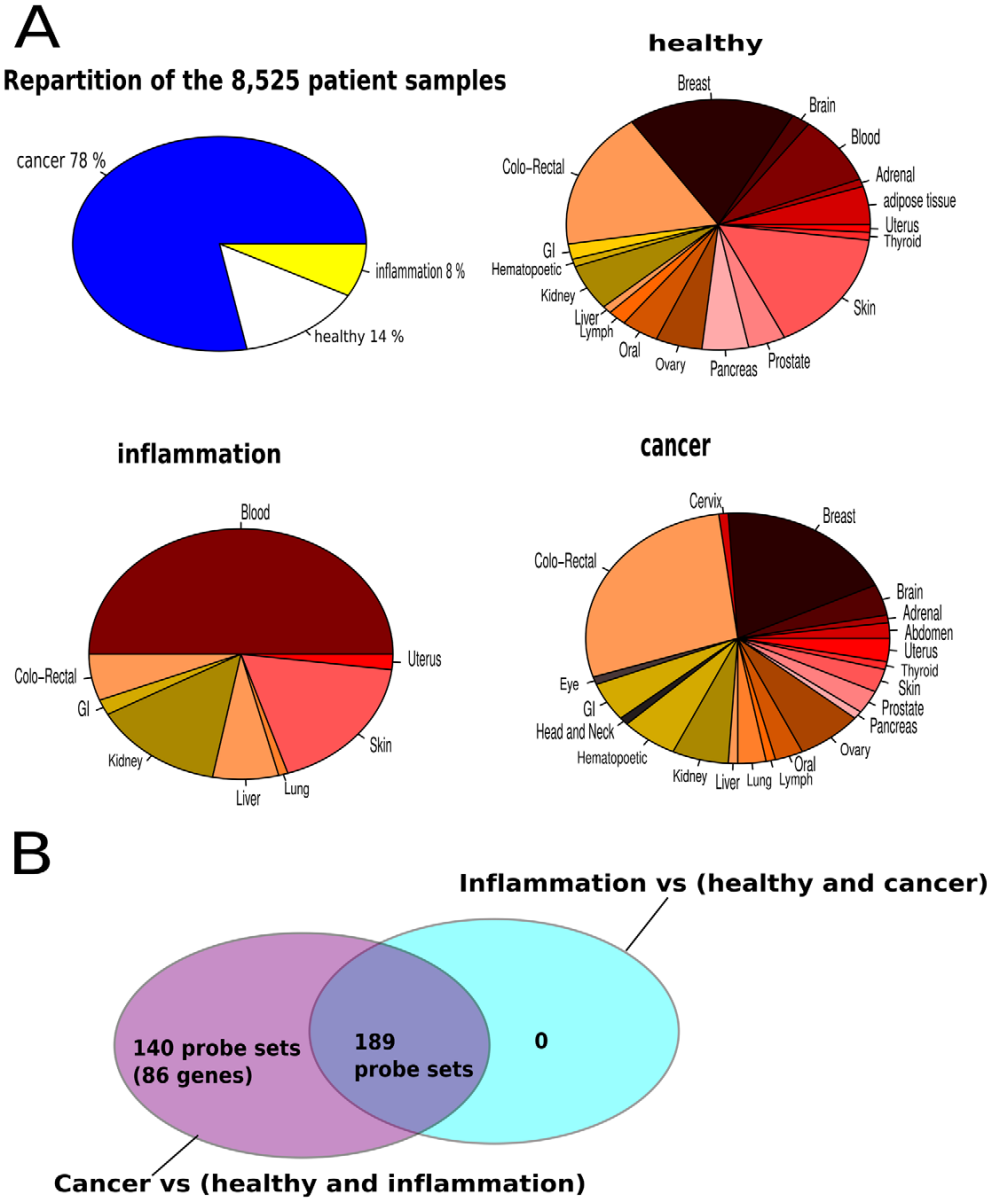
Patient sample repartition and cancer-specific gene expression analysis. Microarray gene expression data representing 8,525 patients samples were downloaded from GEO. A- 78% of patients had different cancer types; 14% are healthy individual and were sampled from different tissues; 8% of patients had inflammation/sepsis and were investigated from the whole blood and other tissues. B- differential expression of the MCN top ten central TFs target gene list coding for secreted and transmembrane proteins were analyzed. Among these genes, as shown in the Venn-diagram, 140 probe sets (86 unique genes) were found to be cancer-specific. GI: Gastro-intestinal.

We further filtered this gene list using available patient data. We performed gene expression analysis on an assembled microarray large data set of 8,525 different tissues from patients with cancer or inflammation and healthy individuals (Figure 4, File S2). From the prioritized 157 genes, we could establish a list of 86 cancer-specific transcripts (Figure 4). Among them, 3 genes were approved by FDA for cancer diagnosis, including: EGFR, KLK3 (PSA) and AFP for the diagnosis of colon, prostate and testis cancers, respectively [43]. Moreover, HLA-F in this list has already been reported as detectable in the serum of various cancer patients using indirect ELISA [10].

### Potential Biomarkers Common in Cancer

**Figure 5:**
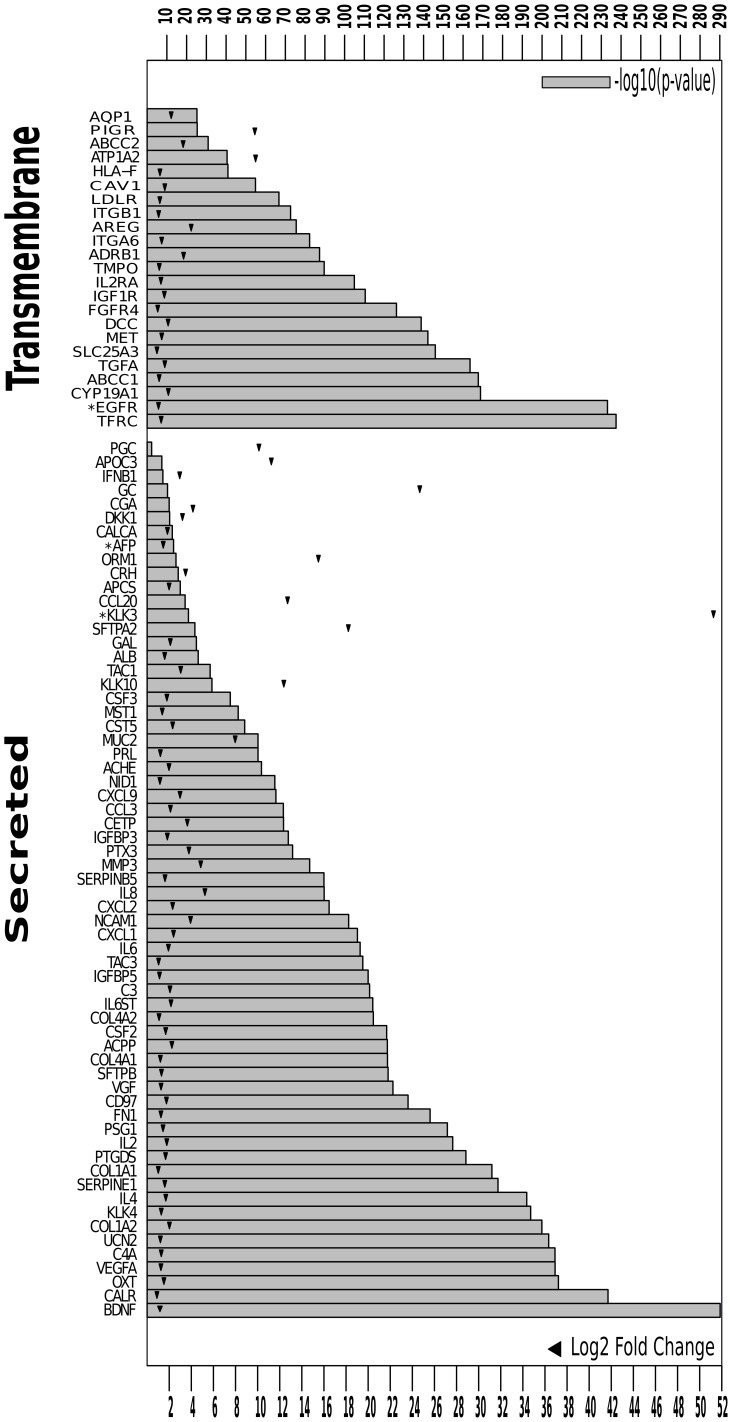
Significant cancer-specific biomarkers from patient gene expression analysis. Cancer-specific gene expression significance and fold change. Significance was attested by B-H p-value correction, and all shown genes have B–H p-value< = 0.05. Bar-plots show the −log10(uncorrected p-value). Triangles show the logged fold change of the corresponding gene in cancer compared to healthy and inflammation patient phenotypes. FDA approved cancer biomarkers are marked with (*).

In order to strengthen the likelihood to find potential common biomarkers among the cancer-specific gene list (Figure 5), we filtered these genes based on their significant effect on patient survival in any of the cancer types from TCGA database. The available gene expression data sets from TCGA, covering nine cancer types, were downloaded and analyzed separately for gene-survival association. For each gene, patients were divided in three groups (tertiles) according to the expression levels of the studied gene. Groups of patients with low, intermediate and high expression were then obtained. Making use of the available patient survival data: follow up duration and death status, we fitted Kaplan-Meier curves to these groups. Genes predicting patient survival significantly (log-rank p-value < = 0.05), in at least one cancer type, are shown in Table S2. The products of these 50 genes mediate many interacting pathways in cancer, as depicted in Figure S2 (KEGG pathway enrichment, p-value ∼4.29E−4).

For each gene listed in Table S2, we added the following resources: (i) CNV significantly affecting the corresponding gene loci in all tumor types as analyzed in the Tumorscape database [44]; (ii) percentage of immunohistochemical (IHC) positive staining in cancer as detected in ProteinAtlas database [45]. We considered that genes positive for all criteria listed in Table S2 are more likely to be common cancer biomarker candidates. TFRC, VEGFA and MET are the best potential candidates. These genes have been separately associated to many cancer types in literature (Table S3).

## Discussion

Cancer types have been screened separately for biomarker identification. Nowadays, there is an emerging effort to search for universal cancer markers. The recently available high-throughput data from cancer patient specimens make this task more affordable in the context of integrative analysis. This study was done within such a framework.

Cancer is a multistage disease, in which normal cells are progressively transformed to malignant ones. This process involves transcription factor (TF) regulation to ensure the transcription of needed genes [46]. We assumed that TFs regulated in cancer would have their activity explained by their coding gene expression level and genomic alterations. We hypothesized that cancer-associated TFs could interact together in a modular manner, such that cancer-triggering events end up perturbing the function of this module. Biomarkers common to many cancer types could be among these TF target genes. We then followed the workflow depicted in Figure 1 to target important genes commonly regulated in cancer that encode accessible proteins. We assumed that focusing on TFs will guide us to find the most valuable part of cancer information, which could be measured by gene expression [47]. Adding CNV data to filter important TFs will strengthen this approach. Whereas, analyzing all regulated genes and significantly altered chromosomal regions without any contextualization in terms of regulators (TFs) will dilute the common cancer biomarker among many false positive outcomes.

As a first step in our quest for common cancer biomarkers, we tried to identify the minimal connected network involving TFs, the activity of which is regulated in tumors. We integrated genomics and transcriptomics data from a panel of cancer cell lines, together with inferred TF regulation from gene expression using TFactS, which has been shown previously to be able to infer accurately TF regulation or activity status from a list of expressed genes [30]. The use of cell lines in this step is justified by the availability of both genomic and expression data. In addition, building meaningful MCN requires data from homogeneous cells, which is not the case of most primary cancer samples, in which genomic alterations and gene expression differ between cancer cells and stromal cells, and even between different cancer cell clones. We identified 88 TFs, which could be the main regulators in cancer cell lines. This step is, however, limited by the TFs represented in TFactS, although they sample the most studied TFs in literature. This step could also be ameliorated by taking into account other genomic alterations, such as mutations. However, whole genome alteration data were not available yet for all the studied cell lines.

By protein-protein interaction analysis, MCN connecting the majority of the 88 TFs has been identified from the curated human proteome network. The MCN contains both TFs and other proteins. Enrichment analysis revealed that this MCN assembles major known pathways driving multiple cancer types. Strikingly, immune response pathways were also enriched in MCN, which was identified based on cell line data, discarding any tumor micro-environmental effect on these results. This suggests a dual role played by this module of connected TFs in both cancer and inflammation. Results from our negative control procedure suggested that the cancer-associated MCN forms a significant module. This module’s most central TFs are susceptible to act as the main “collectors” of marginal perturbations.

In a second step, we arbitrarily limited our analysis to target genes of the top ten most central MCN TFs. Enrichment analysis of these genes revealed a cancer context pathways over-representation, as expected. Since our purpose was to identify genes that could be easily probed in patients we filtered this gene list to 157 genes coding for secreted and transmembrane proteins. By comparing their expression in a panel of 8,525 patients, we identified a set of 86 cancer-specific genes differentially expressed in cancer versus normal and inflammation phenotypes. They include three out of six proteins approved by FDA in specific cancer diagnosis: PSA/KLK3, EGFR and AFP. Expression of these three genes could be checked in other cancer types. PSA, prostate specific antigen, for instance, although widely used in prostate cancer diagnosis, it was also reported in kidney, stomach and breast cancers [48]–[50]. These results provide an internal validation of our methodology.

We sought to further restrict the analysis by taking into account the potential prognostic value in at least one cancer type. This was performed by associating gene expression to patient survival in TCGA data sets. 50 genes significantly predicted survival in at least one cancer type. Each of these genes could be investigated separately in the corresponding cancer type for prognosis. These genes are significantly involved and interconnected in many cancer pathways (Figure S2). Nevertheless, immunomodulatory cytokines and chemokines were also enriched in this gene list, which might suggest that some of these genes may not fully distinguish patients with cancer from those with inflammatory diseases.

We identified three potential biomarkers common to cancer, i.e. TFRC, VEGFA and MET as evidenced by: (i) gene over-expression in cancer compared to normal and inflammation; (ii) gene expression significantly linked to patient survival in at least two cancer types; (iii) corresponding CNV focally significantly amplified in tumors; (iv) proteins stain positive in more than 80% of cancers. VEGFA promotes angiogenesis. Its diagnostic potential was investigated separately in many cancer types (Table S3). MET, is a known oncogenic tyrosine kinase receptor for hepatocyte growth factor. It is also associated with many cancer types (Table S3). In addition, it has been reported as a marker for cancer stem cells in: prostate, head and neck, liver, brain and lung cancers [51]–[56]. VEGFA and MET synergy in angiogenesis might be targeted for more effective anti-tumoral therapy [57]. TFRC, transferrin receptor, is known to be expressed in many tumor types (Table S3). Expression of VEGFA and TFRC is commonly regulated by HIF and MYC, which promote angiogenesis and proliferation, respectively [58]–[60]. The connection between these two TFs via their target genes is known to confer a metabolic advantage to tumors under hypoxia, which is a common condition in malignant diseases [61], [62].

In summary, our strategy identified a network of TFs that regulate 50 potential common cancer biomarkers. Currently available data in TCGA, Tumorscape and ProteinAtlas databases pointed to VEGFA, TFRC and MET genes as potential candidates. Literature knowledge associated to these genes corroborates our approach. Taken together, all these observations might suggest to further investigate the usefulness of VEGFA, MET and TFRC as common cancer biomarkers. This could be performed by direct detection of these biomarkers or by checking for the presence of auto-antibodies directed against potential cancer proteins in patient serum, an approach that has gained much interest in the cancer diagnosis field [4], [63].

## Materials and Methods

### Microarray Analysis

The data from 950 microarrays performed by GlaxoSmithKlein laboratories (GSK) on different cancer cell lines were downloaded from arrayExpress (E-MTAB-37). The RMA normalization method was applied using the xps package from R/Bioconductor [64]. Gene expression on each cell line was performed on duplicates or triplicates. Kolmogorov-Smirnov’s test was performed to select genes differentially expressed in each cell line compared to others. A Bonferroni correction threshold was applied on p-values. Genes with an e-value < = 10 were considered as significantly differentially expressed on the corresponding cell line.

### Transcription Factor Regulation Analysis

Each gene list regulated in each cell line was submitted to TFactS to predict regulated TFs [30]. TFactS sign less catalogue (version 2) contains 6,823 regulations linking 345 unique TFs to their 2,650 unique gene targets. For each list of regulated genes, TFactS predicts the TFs whose targets are enriched in the submitted lists using Fisher’s test. In this study, the larger sign-less catalogue was used instead of the restricted sign-sensitive one. TFactS was executed using BatchTFactS default parameters (www.tfacts.org). TFs with a positive e-value score (−log10(e-value)) were considered as significant. TFs that were not significant in all cell-lines were discarded before the model fitting.

### Genomic Copy Number Variation Analysis

The genomics data of the above cell lines were also released by GSK. SNP arrays data sets available on arrayExpress were downloaded (E-MTAB-38). They were analyzed using the aroma-affymetrix package on R/Bioconductor [65]. Briefly we applied a quantile normalization followed by the CRMA summarization and corrected for chip and PCR fragment length effects [66]. Then the GLAD algorithm was applied to raw copy numbers for segmentation [67]. The segmented data were then submitted to GISTIC algorithm to find significantly altered regions in all chromosomes except X and Y. A default q-value threshold of 0.25 was used to select significant regions [35]. Prior to CNV-based correlation matrix computing and the model fitting, CNV values for each gene in the significantly altered chromosomal regions were normalized as follows: (i) for each of the GISTIC-reported significant regions, we determined the median value of the significant CNV peaks; (ii) each gene in a significant chromosomal region has been assigned the value of this median. The values of the CNV were in log2-ratio as outputted by GISTIC. The chromosomal location of genes was obtained using the Ensembl genes 64 database with human “GRCH37.p5” release in Biomart web tool [68].

### Identification of the Minimal Connected TF Regulated in Cancer Cell Lines

In order to identify a set of correlated TFs that are commonly regulated in cancer, we considered 305 cell lines, for which both expression and SNP data were available. Each TF has three measurements in each cell line: TF regulation scores estimated by TFactS (−log10(e-value)), TF-encoding gene expressions (from microarrays) and TF-locus copy number variations (from median normalized GISTIC analysis). Three matrices, with TFs in rows and cell-lines in columns, could be built from these data: a TF regulation matrix, a TF-encoding gene expression matrix and a TF-locus CNV matrix. In each of these matrices, we computed correlations of each TF with the other TFs using Pearson correlation coefficient. These correlations could be represented as TF-TF correlation profiles. Then we fitted the following model for each TF: R  =  β_0_+ β_1_*E + β_2_*C, Where:

(R) TF-TF correlation profile based on TFactS scores, only TFs significantly regulated in at least one cell line were used; (E) TF-TF correlation profile based on gene expression; (C) TF-TF correlation profile from significant regions identified by GISTIC algorithm, these correlations were computed using loci copy number variation median-normalized values.

Each TF having significant *β_1_* (p-value < = 0.05) and *β_2_* (p-value < = 0.05) was considered as correlatively regulated in cancer. For these TFs, there is an effect of gene expression and genomic alteration (CNV) on their regulation.

The list of these significant TFs was submitted to SNOW web tool to identify their minimal connected network [38]. The SNOW parameters were set as follows: curated human protein-protein interactome with at least two experimental evidences, 1,000 random networks for significance, only one extension interaction was allowed to connect the submitted proteins. A built-in mapping was performed by Snow between submitted gene names and the network protein names.

### MCN Protein-protein Interaction Network Analysis

Network analysis was done using Cytoscape [69]. MCN node centrality analysis was computed using CentiScaPe plugin. Proteins were ranked according to highest betweenness [39]. Target genes of the top ten ranked TFs were kept for further analysis.

### MCN Negative Control

From the list of all TFs represented in TFactS sign-less catalogue, we generated 100 random lists containing 88 distinct elements. These 100 random lists were submitted to Snow web tool using the same parameters as above. The betweenness distribution of the identified cancer-related MCN was compared to the distribution of the whole random MCNs using Kolmogorov-Smirnov’s test in R. The betweenness scores used here are from Snow output.

### Functional Enrichment Analysis

The DAVID web tool was used to perform a functional analysis of the selected target genes [70]. A p-value threshold of 0.05 was used for significance.

### Cancer Patients Gene Expression Analysis

Data from 8,525 patient samples analyzed with HG-U133A2Plus microarrays were downloaded from GEO database. Patient categories were distributed as follows: 78% cancer, 8% inflammation and 14% healthy. Expression data were log2-transformed then normalized per gene by subtracting the gene median expression and dividing by the inter-quartile-range of its expression vector. This data set was used to compare gene expression profiles of genes significantly annotated as coding for secreted or transmembrane proteins using SP-PIR annotation keywords on DAVID from the known targets of the MCN top ten central TFs. Genes significantly differentially expressed were determined between cancer compared to inflammation and healthy phenotypes on one hand, and between inflammation compared to cancer and healthy phenotypes on the other hand. Genes specific to cancer were significantly differentially regulated in the former comparison but not in the later. Differentially regulated genes were computed using R/Bioconductor (limma package) and significance was controlled using B–H correction of p-value < = 0.05 [64], [71]. The cancer-specific gene list identified from this analysis was considered as the accessible cancer-specific biomarker list (genes specific to cancer and coding for secreted and transmembrane proteins).

### Survival Analysis

The accessible cancer-specific biomarker list was evaluated for prognosis potential in different TCGA published cancer data sets. TCGA database offers a set of gene expression from clinically annotated patient samples (http://tcga-data.nci.nih.gov/tcga/). We downloaded (09/27/2011) level2 gene expression data for all the patients from 9 publicly available cancer types (GBM: Glioblastoma multiform, OV: Ovarian serous cystadenocarcinoma, LAML: Acute Myeloid Leukemia, BRCA: Breast invasive carcinoma, COAD: Colon adenocarcinoma, KIRC: Kidney renal clear cell carcinoma, LUSC: Lung squamous cell carcinoma, UCEC: Uterine Corpus Endometrioid Carcinoma). The number of patients per gene expression data set was distributed as follows: BRCA: 600; COAD: 179; GBM: 536 (agilent) or 555 (affy); KIRC: 72; LAML: 197; LUSC: 161 (agilent) or 134 (affy); OV: 608 (agilent) or 590 (affy); READ: 78; UCEC: 54. Survival data were among the clinical information available for the majority of these patients. For each gene, in each cancer type, the expression vector from level2 TCGA data was standardized by median subtraction and inter-quartile range division. Then each normalized expression vector corresponding to each gene was divided on three groups (tertiles). Patients in the lower tertile were assigned a gene down-regulation; patients in the intermediate tertile were assigned an intermediate gene expression; patients in the upper tertile were assigned an up-regulation of the gene in interest. The R/Bioconductor (survival package) was used to fit survival curves on categorized patients for each gene [72]. Three Kaplan-Meier survival curves were then fitted for each gene corresponding to up-regulation, intermediate and down-regulation groups of patients according to their death status and follow up duration. Significance of the difference between these curves was estimated based on the log-rank p-value < = 0.05. If data from more than one platform was available (Affymetrix or Agilent), the analysis is done independently on each.

## Supporting Information

Figure S1 **Copy Number Variation results.** Copy Number Variation GISTIC scores and q-values for the whole cancer cell-lines data set. Significance profiles (q-values in the bottom and scores in the top horizontal axes) are shown for chromosomal regions in the left and right vertical axes of the panels for : A- amplified regions, B- deleted regions.
(TIF)

Figure S2 **Patient survival-affecting genes are involved in many cross-talking cancer pathways.** Genes significantly affecting cancer patient survival are mechanistically interacting to trigger important cancer pathways. These genes are enriched (p-value  = 4.29E−4) in KEGG’s cancer pathways analyzed by DAVID web-tool.
(TIF)

File S1 **Enrichment analysis of the top 10 MCN TF target genes**
(XLS)

File S2 **Patient gene expression data sets from GEO database and their accession numbers.**
(XLS)

Table S1 **Transcription factors regulated in cancer cell lines.** For each TF, the TF-TF correlations profiles were determined and the following model was fitted: reg  =  β_0_+ β_1_ * exp + β_2_ * cnv. Where, for each TF versus the others: “reg” is the regulation correlation profile, “exp” is the TF-coding gene expression correlation profile and “cnv” is the TF coding-gene locus copy number variation correlation profile.
(PDF)

Table S2 **Filtered cancer-specific genes.** Significant cancer-specific genes identified in patient microarray analysis were filtered to those that are significantly associated (p-value < = 0.05) with at least one cancer type survival based on TCGA data. The resulting gene list is shown here. Tumorscape database gives q-values for CNV alterations in a large collection of tumors. Based on the CNV from all tumors pooled together, amplification and deletion q-values corresponding to the chromosomal locations of these genes are shown. Whether the loci is focally affected by the CNV is also shown. ProteinAtlas database contains cancer and normal tissue IHC staining for proteins with available antibodies. The percentage of staining in cancer is shown. N.A: not available, N.S: non-significant; GBM: Gliobastoma mutiforme, OV: Ovarian serous cystadenocarcinoma, LAML: Acute Myeloid Leukemia, BRCA: Breast invasive carcinoma, COAD: Colon adenocarcinoma, KIRC: Kidney renal clear cell carcinoma, LUSC: Lung squamous cell carcinoma, UCEC: Uterine Corpus Endometrioid Carcinoma. K-M: Kaplan-Meier log-rank test.
(PDF)

Table S3 **Evidences of VEGFA, TFRC and MET associations to cancer from a non-exhaustive literature screening.**
(PDF)
