# Supplementary figures and images for: A Minimal Connected Network of Transcription Factors Regulated in Human Tumors and Its Application to the Quest for Universal Cancer Biomarkers
Source: PLoS One. 2012 Jun 25;7(6):e39666. doi: 10.1371/journal.pone.0039666 (PMC3382591; doi:10.1371/journal.pone.0039666)

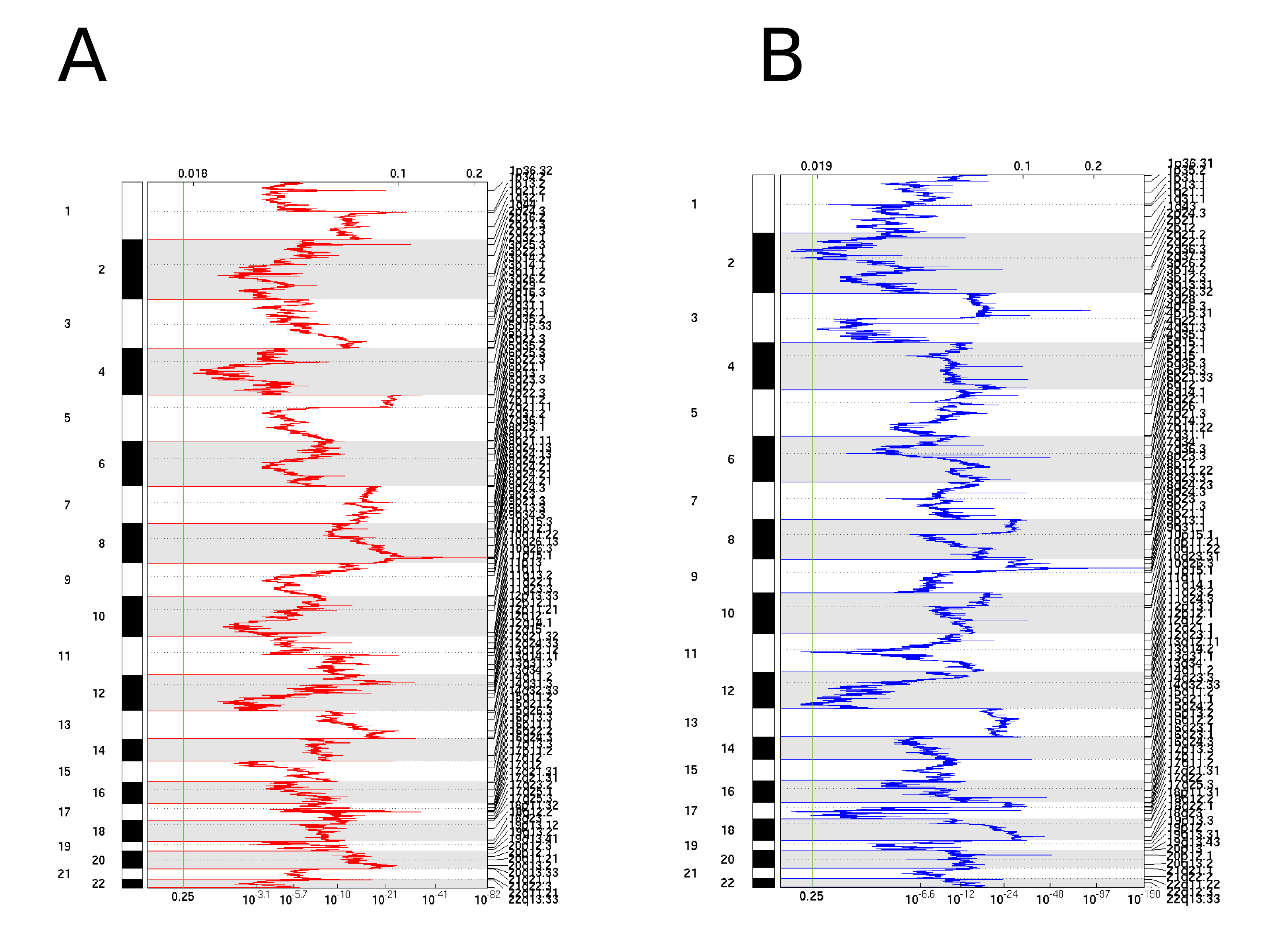

Supplement: Figure S1 — Copy Number Variation results. Copy Number Variation GISTIC scores and q-values for the whole cancer cell-lines data set. Significance profiles (q-values in the bottom and scores in the top horizontal axes) are shown for chromosomal regions in the left and right vertical axes of the panels for : A- amplified regions, B- deleted regions. (TIF) [file pone.0039666.s001.tif]

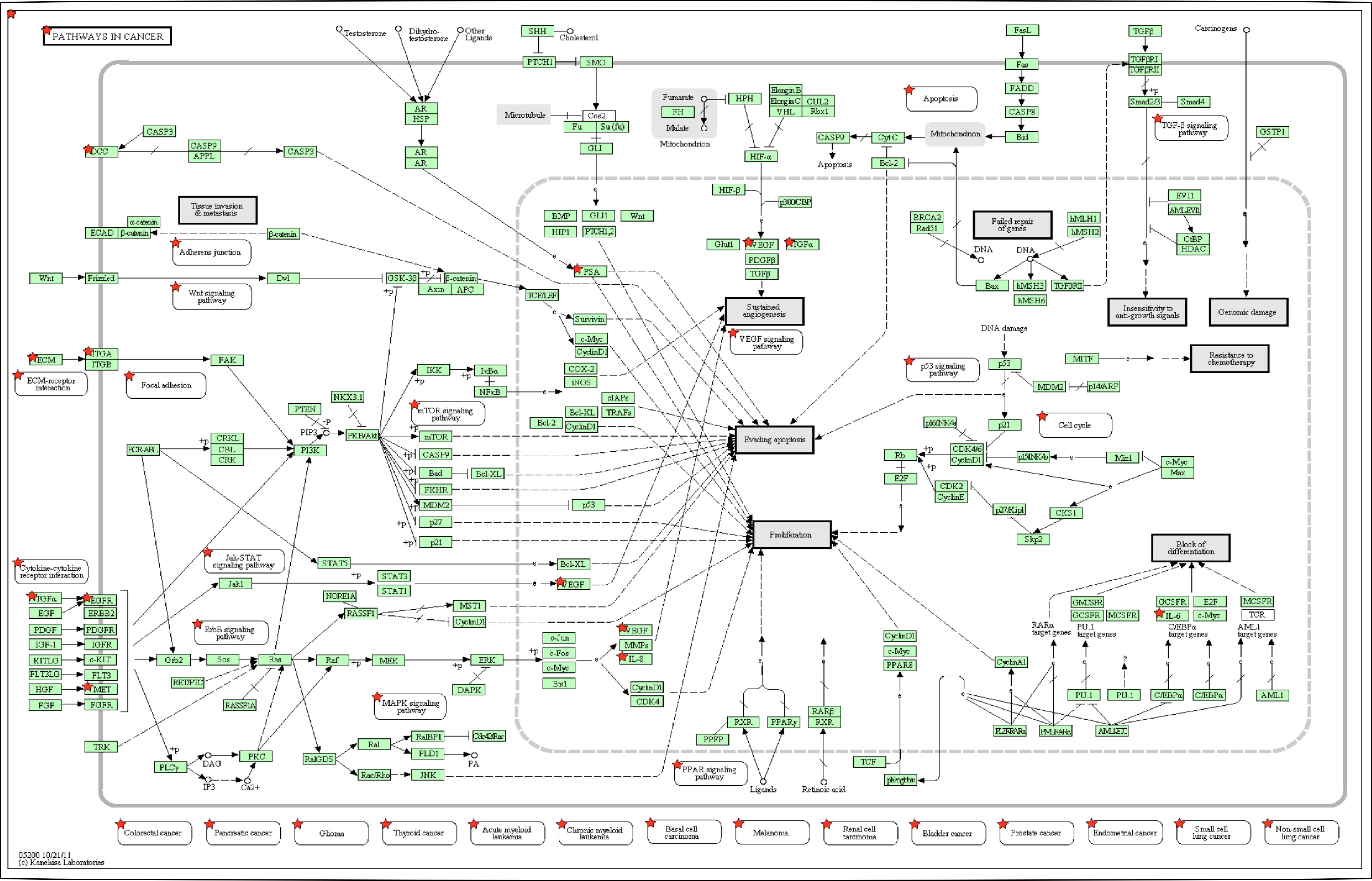

Supplement: Figure S2 — Patient survival-affecting genes are involved in many cross-talking cancer pathways. Genes significantly affecting cancer patient survival are mechanistically interacting to trigger important cancer pathways. These genes are enriched (p-value = 4.29E−4) in KEGG’s cancer pathways analyzed by DAVID web-tool. (TIF) [file pone.0039666.s002.tif]
