# Supplementary material for: A Minimal Connected Network of Transcription Factors Regulated in Human Tumors and Its Application to the Quest for Universal Cancer Biomarkers
Source: PLoS One. 2012 Jun 25;7(6):e39666. doi: 10.1371/journal.pone.0039666 (PMC3382591; doi:10.1371/journal.pone.0039666)
Supplement: Table S1 — Transcription factors regulated in cancer cell lines. For each TF, the TF-TF correlations profiles were determined and the following model was fitted: reg = β0+ β1 * exp + β2 * cnv. Where, for each TF versus the others: “reg” is the regulation correlation profile, “exp” is the TF-coding gene expression correlation profile and “cnv” is the TF coding-gene locus copy number variation correlation profile. (PDF) [file pone.0039666.s005.pdf]

Table S1: Transcription factors regulated in cancer cell lines

*For each TF, the TF-TF correlations profiles were determined and the following model was fitted: reg ~ β_0 + β_1 * exp + β_2 * cnv. Where, for each TF versus the others: "reg" is the regulation correlation profile, "exp" is the TF-coding gene expression correlation profile and "cnv" is the TF coding-gene locus copy number variation correlation profile.*

**TF**	**Expression correlation effect (β_1 p-value)**	**CNV correlation effect (β_2 p-value)**
TP53	2.56E-12	4.76E-02
ONECUT1	9.98E-10	1.26E-02
RARA	5.44E-09	2.33E-02
PGR	7.19E-09	4.39E-04
STAT1	9.71E-09	3.75E-04
CREB1	1.28E-08	1.58E-02
PITX2	4.03E-08	3.82E-03
CREBBP	1.37E-07	1.91E-04
POU1F1	4.06E-07	4.65E-02
PPARA	4.70E-07	1.45E-03
FOXA2	5.74E-07	2.03E-03
SMAD1	6.29E-07	1.16E-02
ATF6	7.90E-07	1.12E-02
CEBPG	8.89E-07	2.53E-02
SOX10	1.51E-06	7.52E-03
ETS1	2.03E-06	2.77E-03
MYBL1	2.09E-06	6.76E-03
SREBF1	2.20E-06	4.69E-02
MYC	2.44E-06	3.98E-03
E2F6	3.68E-06	2.36E-02
ZBTB16	3.89E-06	8.09E-06
ERG	5.14E-06	5.72E-03
HIF1A	1.05E-05	1.42E-03
PPARD	1.38E-05	2.06E-02
TCF7	2.35E-05	3.23E-04
NFIA	2.40E-05	4.06E-03
SP2	3.72E-05	5.21E-04
POU2F2	4.54E-05	4.26E-02

NR3C1	5.08E-05	1.11E-03
ID1	1.37E-04	3.54E-02
MSX2	1.52E-04	9.45E-03
PAX3	1.60E-04	4.90E-02
FOS	1.63E-04	1.80E-03
REST	1.85E-04	1.81E-06
SMAD7	1.98E-04	3.00E-04
POU5F1	2.43E-04	9.11E-03
NFATC2	2.84E-04	6.62E-03
CEBPE	3.01E-04	1.66E-02
E2F2	4.07E-04	2.02E-02
FOSB	4.10E-04	4.20E-03
EBF1	4.25E-04	8.35E-07
GATA3	4.77E-04	3.15E-02
SMAD6	4.81E-04	6.37E-04
FOXH1	4.89E-04	2.60E-05
GLI1	4.95E-04	1.81E-03
STAT5A	5.81E-04	2.33E-07
GLI2	6.60E-04	4.64E-03
HNF4A	6.70E-04	2.39E-04
CEBPA	6.77E-04	1.59E-03
STAT3	8.08E-04	1.90E-06
LEF1	8.69E-04	8.73E-03
HOXC8	9.56E-04	4.12E-02
NANOG	9.94E-04	3.27E-02
GABPA	9.96E-04	1.18E-02
MEF2A	1.44E-03	1.74E-04
ATF4	1.47E-03	1.32E-02
YY1	1.53E-03	8.75E-03
EPAS1	1.65E-03	1.94E-02
FOXO1	1.96E-03	2.13E-04
ESR1	2.06E-03	6.92E-04
BRCA1	2.06E-03	3.88E-04
CDX1	2.22E-03	9.86E-04
RARB	2.26E-03	2.07E-02

NR2F1	3.03E-03	4.22E-02
BCL3	3.03E-03	1.92E-02
PDX1	3.35E-03	7.14E-03
HOXA5	3.67E-03	1.31E-02
ESR2	4.00E-03	4.59E-03
STAT5B	4.19E-03	1.45E-07
REL	4.32E-03	3.00E-03
OLIG1	6.07E-03	1.05E-02
NFYA	6.93E-03	8.00E-03
RXRA	8.61E-03	1.71E-04
DLX5	9.38E-03	4.66E-03
NR5A1	9.42E-03	5.55E-03
TRPS1	1.34E-02	1.02E-02
SREBF2	1.52E-02	2.63E-03
THRA	1.52E-02	2.38E-02
HOXD3	1.57E-02	1.35E-02
RARG	1.79E-02	1.99E-02
CDKN1A	1.92E-02	4.65E-05
RBPJ	2.00E-02	2.80E-03
FLI1	2.32E-02	9.55E-06
E2F4	2.46E-02	5.89E-04
RUNX1	2.77E-02	9.26E-04
TEAD1	3.43E-02	1.81E-02
NFKB2	4.46E-02	1.02E-02
SP4	4.60E-02	1.35E-03
