# Supplementary material for: A Minimal Connected Network of Transcription Factors Regulated in Human Tumors and Its Application to the Quest for Universal Cancer Biomarkers
Source: PLoS One. 2012 Jun 25;7(6):e39666. doi: 10.1371/journal.pone.0039666 (PMC3382591; doi:10.1371/journal.pone.0039666)
Supplement: Table S2 — Filtered cancer-specific genes. Significant cancer-specific genes identified in patient microarray analysis were filtered to those that are significantly associated (p-value < = 0.05) with at least one cancer type survival based on TCGA data. The resulting gene list is shown here. Tumorscape database gives q-values for CNV alterations in a large collection of tumors. Based on the CNV from all tumors pooled together, amplification and deletion q-values corresponding to the chromosomal locations of these genes are shown. Whether the loci is focally affected by the CNV is also shown. ProteinAtlas database contains cancer and normal tissue IHC staining for proteins with available antibodies. The percentage of staining in cancer is shown. N.A: not available, N.S: non-significant; GBM: Gliobastoma mutiforme, OV: Ovarian serous cystadenocarcinoma, LAML: Acute Myeloid Leukemia, BRCA: Breast invasive carcinoma, COAD: Colon adenocarcinoma, KIRC: Kidney renal clear cell carcinoma, LUSC: Lung squamous cell carcinoma, UCEC: Uterine Corpus Endometrioid Carcinoma. K-M: Kaplan-Meier log-rank test. (PDF) [file pone.0039666.s006.pdf]

Table S2: Filtered cancer-specific genes

Significant cancer-specific genes identified in patient microarray analysis were filtered to those that are significantly associated (p-value <= 0.05) with at least one cancer type survival based on TCGA data. The resulting gene list is shown here. Tumorscape database gives q-values for CNV alterations in a large collection of tumors. Based on the CNV from all tumors pooled together, amplification and deletion q-values corresponding to the chromosomal locations of these genes are shown. Whether the loci is focally affected by the CNV is also shown. ProteinAtlas database contains cancer and normal tissue IHC staining for proteins with available antibodies. The percentage of staining in cancer is shown. N.A: not available, N.S: non-significant; GBM: Gliobastoma mutiforme, OV: Ovarian serous cystadenocarcinoma, LAML: Acute Myeloid Leukemia, BRCA: Breast invasive carcinoma, COAD: Colon adenocarcinoma, KIRC: Kidney renal clear cell carcinoma, LUSC: Lung squamous cell carcinoma, UCEC: Uterine Corpus Endometrioid Carcinoma. K-M: Kaplan-Meier log-rank test.

**gene name**	**Cancer significance p-value**	**TCGA cancer type survival-associated (K-M p-value <= 0.05)**	**proteinAtlas cancer IHC staining %**	**Tumorscape all cancer amplification q-value**	**amplification focal?**	**Tumorscape all cancer deletion q-value**	**deletion focal?**
BDNF	6.76E-291	GBM; OV	91.00%	NS	N.A	NS	N.A
**TFRC**	**3.42E-238**	**GBM; LUSC**	**82.00%**	**1.94E-08**	**Yes**	NS	N.A
CALR	4.10E-234	OV	81.00%	NS	N.A	NS	N.A
EGFR	7.66E-234	OV	44.00%	1.57E-46	Yes	NS	N.A
**VEGFA**	**2.58E-207**	**LUSC; OV; GBM**	**100.00%**	**5.46E-07**	**Yes**	NS	N.A
COL1A2	1.26E-200	OV	N.A	2.46E-09	Yes	NS	N.A
KLK4	6.13E-195	LAML	N.A	NS	N.A	1.47E-04	No
IL4	5.88E-193	GBM; LUSC	N.A	NS	N.A	2.81E-05	Yes
SERPINE1	2.09E-178	GBM	14.00%	4.53E-03	No	NS	N.A
TGFA	2.94E-164	GBM	79.00%	NS	N.A	NS	N.A
IL2	1.76E-155	OV	55.00%	NS	N.A	2.60E-18	No
PSG1	9.20E-153	LAML	N.A	NS	N.A	NS	N.A
SLC25A3	1.16E-146	OV	N.A	NS	N.A	1.23E-26	No
FN1	5.07E-144	OV	52.00%	NS	N.A	2.55E-04	No
**MET**	**6.63E-143**	**GBM; LAML**	**99.00%**	**1.25E-07**	**Yes**	6.96E-03	No
DCC	1.65E-139	LAML	99.00%	NS	N.A	1.10E-01	Yes
CD97	7.22E-133	GBM	77.00%	NS	N.A	NS	N.A
FGFR4	5.38E-127	LUSC	68.00%	5.93E-05	Yes	4.82E-02	No
VGF	3.22E-125	COAD	N.A	7.46E-03	No	NS	N.A
CSF2	5.00E-122	GBM	N.A	NS	N.A	4.28E-07	Yes
IL6ST	5.42E-115	BRCA; GBM; KIRC; LAML; LUSC	40.00%	NS	N.A	2.06E-12	No

C3	2.20E-113	GBM		NS	N.A	5.41E-05	No	
IGFBP5	1.20E-112	GBM	90.00%	NS	N.A	4.56E-05	No	
TAC3	6.05E-110	COAD; GBM	N.A	6.69E-07	No	NS	N.A	
IL6	1.36E-108	KIRC; GBM	3.00%	NS	N.A	2.06E-12	No	
IL2RA	1.15E-105	GBM; LAML	3.00%	NS	N.A	NS	N.A	
NCAM1	8.85E-103	BRCA; LUSC; OV	18.00%	NS	N.A	5.82E-19	Yes	
CXCL2	6.95E-93	BRCA	N.A	NS		NS	N.A	
TMPO	2.21E-90	GBM	95.00%	NS		NS	N.A	
IL8	2.26E-90	GBM	N.A	NS		1.67E-04	No	
SERPINB 5	2.82E-90	BRCA; GBM	75.00%	NS		1.54E-01	No	
ADRB1	4.84E-88	LAML; OV	N.A	NS		3.29E-02	No	
ITGA6	5.26E-83	KIRC	62.00%	NS		NS	N.A	
AREG	3.10E-76	LAML	90.00%	NS		NS	N.A	
PTX3	2.20E-74	GBM	79.00%	2.25E-02	No	NS	N.A	
IGFBP3	3.19E-72	GBM	59.00%	NS		NS	N.A	
LDLR	1.75E-67	LUSC; OV; GBM		NS		8.65E-21	No	
ACHE	1.24E-58	COAD; LUSC	96.00%	4.99E-04	No	NS	N.A	
MUC2	7.03E-57	BRCA; GBM; READ	19.00%	NS		1.08E-07	Yes	
CAV1	1.31E-55	GBM	26.00%	8.26E-06	No	NS	N.A	
HLA-F	1.18E-41	OV; READ; GBM	N.A	NS		NS	N.A	
ATP1A2	3.23E-41	LUSC	10.00%	1.11E-16	No	NS	N.A	
KLK10	1.50E-33	BRCA	51.00%	NS		6.09E-04	No	
PIGR	4.31E-26	BRCA; GBM; READ	39.00%	NS		NS	N.A	
AQP1	5.69E-26	COAD; GBM; OV	24.00%	NS		4.17E-37	No	
GAL	1.12E-25	GBM	76.00%	1.06E-32	No	1.09E-40	No	
KLK3	1.17E-21	LAML	5.00%	NS		2.23E-04	No	
CCL20	5.76E-20	BRCA; GBM	N.A	NS		2.80E-09	No	
DKK1	3.86E-12	KIRC	83.00%	NS		NS	N.A	
IFNB1	1.04E-08	KIRC	100.00%	NS		6.48E-93	No	
