# Supplementary material for: A Minimal Connected Network of Transcription Factors Regulated in Human Tumors and Its Application to the Quest for Universal Cancer Biomarkers
Source: PLoS One. 2012 Jun 25;7(6):e39666. doi: 10.1371/journal.pone.0039666 (PMC3382591; doi:10.1371/journal.pone.0039666)
Supplement: Table S3 — Evidences of VEGFA, TFRC and MET associations to cancer from a non-exhaustive literature screening. (PDF) [file pone.0039666.s007.pdf]

Table S3: Evidences of VEGFA, TFRC and MET associations to cancer from non-exhaustive literature screening

Protein	***cancer type***	***Pubmed reference ids***
VEGFA	Liver cancer	21802642; 12810638
	Skin cancer	21284506; 12170181
	Colo-rectal cancer	21081932
	malignant gynecological diseases	20442584
	Ovarian cancer	20071014
	Brain cancer	19661350; 19522573
	Lung cancer	19157636; 17917830; 20357617
	Pancreatic cancer	18665074
	Thyroid Cancer	11932308
TFRC	Lymphoma	12218295; 6131211
	Leukemia	2582441
	Breast cancer	3024390
	Colo-rectal cancer	9735419
	Pancreatic cancer	15177502
	Bladder cancer	2340368
	Brain cancer	2110696; 14756479
	Prostate cancer	15514585
	Liver cancer	2836292
	Lung cancer	2414490
	Skin cancer	3549812
	Oral cancer	1607823
MET	Thyroid Cancer	17703498
	Ovarian cancer	21945764
	Bladder cancer	16400012
	Liver cancer	21955323
	Breast cancer	21692045
	Prostate cancer	19549766
	Gastric cancer	21777278
	Pancreatic cancer	20869080
